# Upregulated Long Noncoding RNA UCA1 Enhances Warburg Effect via miR-203/HK2 Axis in Esophagal Cancer

**DOI:** 10.1155/2020/8847687

**Published:** 2020-11-04

**Authors:** Hai-E Liu, Hao-Hong Shi, Xing-Jing Luo

**Affiliations:** ^1^Department of Anesthesia, Children's Hospital of Fudan University, Shanghai, China; ^2^Department of Anesthesia, Anhui Provincial Children's Hospital, Hefei, Anhui, China

## Abstract

Reprogrammed glucose metabolism of enhanced aerobic glycolysis, also known as Warburg effect, which exerts a significant contributor to cancer progression, is regarded as a hallmark of cancer. The roles of long noncoding RNAs (lncRNA) in regulating cancer via metabolic reprogramming are mostly unknown, including esophagal cancer (EC). Here, we showed that how the lncRNA urothelial carcinoma associated 1 (UCA1) exerts pro-oncogene in regulating EC glucose metabolism. Firstly, we found that upregulated UCA1 expression enhances the malignant phenotypes of EC, including poor outcome, larger tumor size, positive lymphatic invasion, and advanced pathological stages. UCA1 silencing could suppress EC cell proliferation and metastasis. Following, bioinformatics analyses revealed that UCA1 regulated the HK2 expression through functioning as a competing endogenous RNA (ceRNA). Mechanistically, UCA1 overexpression could elevate the activation of HK2 oncogenes via inhibition of miR-203 activity, as evidenced by the positive correlation of UCA1 with HK2 and inverse correlation with miR-203 expression. Luciferase activity assay further verified the targeting relationship between UCA1, miR-203, and HK2. Upregulated UCA1 in EC cells significantly suppressed the degradation of HK2 by miR-203. Further research showed that upregulated UCA1 effectively increased the rate of glucose uptake, lactate output, and ECAR value, all of which can be attenuate by HK2 interference and 2-DG, whereas knockdown of UCA1 had the opposite effect. In sum, our findings suggest that the UCA1/miR-203/HK2 axis contributes to EC development by reprogramming tumor glucose metabolism, providing new insight into the management of EC patients.

## 1. Introduction

Esophageal cancer (EC) is the eighth most common aggressive malignancies worldwide and ranks sixth most lethal tumor [1, 2]. In China, it is the fourth most frequently diagnosed cancer and ranks as one of the top five deadliest cancers [3]. Even with advances in diagnostic technology, it is difficult to diagnose at early stages for most of the patients, and advanced EC frequently possess an aggressive profile and the clinical manifestations of dysphagia [4, 5]. Despite great advances in EC treatment, including numerous types and combinations of treatments, including surgery, radiotherapy, and chemotherapy as well as other therapeutic strategies, the outcome of EC is still not satisfactory due to local recurrence or distant metastasis [6, 7]. Currently, poor survival rate of EC patients with less than 50%, even between 15 and 25% [8], makes it urgent for uncovering the underlying mechanisms of EC for more effective therapy improvement.

Long noncoding RNAs (lncRNAs), a class of RNAs (>200 nt) without coding capacity resulting from incomplete open-reading frame [9], have been found to play roles in multibiological processes, including X-chromosome inactivation [10], chromatin modification [11], and microRNA sponging [12]. Accumulating studies has pointed out that dysregulation of lncRNAs was associated with various malignant tumors [13], including urothelial carcinoma associated 1 (UCA1). In breast cancer, UCA1 could promote EMT by activating the Wnt/*β*-catenin signaling pathway [14]. In PDAC, increased UCA1 expression was involved in the Hippo pathway to promote tumorigenesis [15]. Besides, UCA1 worked as an oncogene in other cancers, including colorectal cancer, ovarian cancer, and GC [16–18]. However, very little research about the role of UCA1 in EC and its underlying mechanism has been disclosed.

In this study, we investigated the roles and the mechanism undergoing in the EC progress of UCA1. As well as the current study provides the first evidence that UCA1 promotes aerobic glycolysis by suppressing the degradation of HK2 via spongingmiR-203 in EC.

## 2. Materials and Methods

### 2.1. Cell Culture

Four EC cell lines (EC1, EC109, EC9706, and KYSE150) and a human esophageal epithelial cell line (Het-1A) were purchased from the Cell Bank of the Chinese Academy of Sciences (Shanghai, China) and cultivated in RPMI-1640 (Gibco, Grand Island, NY, USA) with 10% fetal bovine serum (FBS, Gibco) at a modified atmosphere with 5% CO_2_.

### 2.2. Patients and Specimens

A total of 110 cancer tissues and 60 paired of adjacent nontumor tissues of which were obtained from the Fourth Hospital of Hebei Medical University from April 2010 to May 2016, who were pathologically and histologically diagnosed as EC. No chemotherapy or radiotherapy was performed before surgery. Tissues were immediately stored at -80°C until use. The study was in line with the standards set by the Declaration of Helsinki. Written informed consents were obtained from all patients. This study was approved by the Institutional Ethical Board of the Fourth Hospital of Hebei Medical University.

### 2.3. RNA Isolation and qRT-PCR

Total RNA was isolated using RNAiso Plus (Takara, Shiga, Japan) in accordance with the manufacturer's protocol. cDNA was synthesized using the RevertAid First Strand cDNA Synthesis kit (Thermo Fisher Scientific). miRNA expression was detected by using the TIANGEN^®^ miRcute PlusmiRNA qPCR Kit (FP411) using U6 as an internal control. The messenger RNA (mRNA) expression was measured by using an SYBR Green qPCR assay (TaKaRa, Dalian, China) using GAPDH mRNA as an endogenous control. Primers for the qRT-PCR were as follows: U6 primer forward, 5′-CTCGCTTCGGCAGCACA-3′ and reverse, 5′-AACGCTTCACGAATTTGCGT-3′; UCA1 primer forward, 5′-TTTGCCAGCCTCAGCTT.

AAT-3′ and reverse, 5′-TTGTCCCCATTTTCCATCAT-3′; GAPDH primer forward, 5′- GACTCATGACCACAGTCCATGC-3,′ and reverse, 5′-AGAGGCAGGGATGATGTTCTG-3′; miR-203 primer forward, 5′- GCGTGAAATGTTTAGGACCACTAG-3′ and reverse, 5′- CGGTAGCTTATCAGACTGATGTTGA-3′. HK2 primer forward, 5′-CAAAGTGACAGTGGGTGTGG-3′ and reverse, 5′- GCCAGGTCCTTCACTGTCTC-3′. The 2^−ΔCt^ method was used to calculate the relative levels of gene expression.

### 2.4. Cell Viability Assay

Cell viability was measured daily using a standard Cell Counting Kit-8 assay (Dojindo, Kumamoto, Japan) according to the manufacturer's instructions. 3000 cells per well were seeded into 96-well plates (100 *μ*l per well).

### 2.5. Immunoblotting

Total protein lysates were isolated with RIPA lysis buffer and separated on 10% SDS-PAGE gel followed by transferring to a PVDF Immobilon-P membrane (Millipore, MA). Primary antibodies used in this study were as follows: rabbit mAb anti-HK2 (1 : 1000, CST) and mouse mAb anti-GAPDH (1 : 1000, CST) were used for western blot analysis. Relative proteins expression levels were normalized to GAPDH levels.

### 2.6. Cell Migration and Invasion Assay

This assay was performed as previously described [19].

### 2.7. Stable Cell Lines

MiR-203 mimics, miR-203 inhibitor, lentiviruses short hairpin (shRNA) directed against UCA1 (sh-MALAT1) and the empty lentiviral vector (sh-NC), pCD513B1 empty (LV-NC), and pCD513B1- UCA1 lentiviruses (LV-uca1) were prepared for transfecting using lipofectamine 2000 (Invitrogen) according to the manufacturer's instructions.

### 2.8. Dual-Luciferase Reporter Assay

Cells were cultured in 96-well plates and transfected with wild-type (WT) or mutated (Mut) 3'-UTR of UCA1 or HK2, together with sh-UCA1, lv-UCA1, miR-203 mimic, anti-miR-203, or corresponding negative control (NC). Forty-eight hours after transfection, luciferase activities were measured by dual-luciferase activity assays (Promega, Madison, WI, USA) according to the manufacturer's instructions.

### 2.9. RNA Immunoprecipitation Assay

RNA immunoprecipitation assay was implemented as previously described [20], and it used the EZ-Magna RIP RNA-binding protein immunoprecipitation kit (Millipore) following manufacturer's instructions.

### 2.10. Glucose Consumption and Lactate Production Assay

The glucose assay kit (ThermoFisher Scientific) and lactate Assay kit (Abcam, ab65330) were used to test glucose consumption and lactate production following manufacturer's instructions, respectively. The values were normalized to total protein concentration.

### 2.11. Detection of ECAR

The Seahorse XF96 flux analyser (Seahorse Bioscience) was used to detect the glycolysis by monitoring the ECAR (extracellular acidification rate), according to the manufacturer's instructions. Briefly, 1 × 10^4^ cells were seeded in a XF 96-well plate overnight. The detection of ECAR was incubated with unbuffered medium followed by injecting sequential inhibitors: 10 mM glucose, 1 mM oligomycin, and 80 mM 2-deoxyglucose (2-DG).

### 2.12. Statistical Analysis

SPSS and GraphPad Prism 5 software were used for statistical analysis. Cumulative survival time was tested by the KaplanMeier method and analyzed by the log-rank test. The data are presented as the mean ± SD with three individuals and analyzed with Student's *t* test. A *p* < 0.05 was considered statistically significant.

## 3. Results

### 3.1. Increased UCA1 Enhances the Malignant Phenotypes of EC

**Figure 1:**
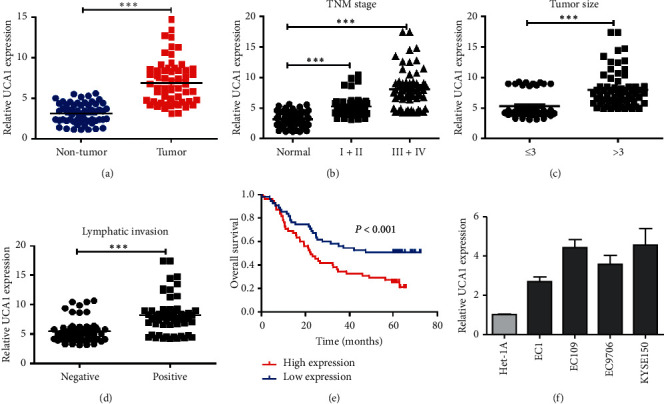
Highly expressed UCA1 existed in EC cells and tissues. (a) Different expression levels of UCA1 between paired cancerous and adjacent noncancer tissues (*n* = 60). (b) UCA1 expression was significantly higher in patients at advanced pathological stages. (c) UCA1 expression was significantly higher in larger tumor size samples. (d) UCA1 expression was significantly higher in sample lymph node metastasis. (e) KaplanMeier survival curves showed the correlation between UCA1 expression and outcome in EC patients (*p* < 0.001). (f) The relative expression of UCA1 in Het1-A cells and 4 EC cell lines was assessed by qRT-PCR. Paired Student's *t* test was used in (a) (c), (d), and (e); ^*∗∗∗*^*p* < 0.001.

To explore the function of UCA1 in ES, the expression of UCA1 in 60 pairs of ES and adjacent nontumor tissues were detected by RT-qPCR. As shown in Figure 1(a), UCA1 expression was significantly higher in tumor tissues than in paired adjacent nontumor tissues, in particular in tissues with advanced pathological stages (Figure 1(b); *p* < 0.001), larger tumor size (Figure 1(c); *p* < 0.001), and positive lymphatic invasion (Figure 1(d); *p* < 0.001). And, the chi-squared test indicated that UCA1 expression in EC tissues was closely related to tumor size (*p*=0.033), alcohol status (*p*=0.004), lymphatic invasion (*p* < 0.001), distant metastasis (*p*=0.001), and TNM stage (*p* < 0.001), whereas no significant differences were found between UCA1 expression and age, gender, smoking status, tumor location, and differentiation (Table 1). Following this, the association of UCA1 expression with the outcome was determined in 110 EC cases. The results showed that higher UCA1 expression indicated unfavorable prognosis using KaplanMeier survival curves and the log-rank test (Figure 1(e)). Then, univariate and multivariate analysis were applied using a Cox proportional hazards model to analyze the relationship of UCA1 expression with clinical parameters and patients' outcomes. Univariate and multivariate Cox analysis demonstrated that UCA1 expression, lymphatic invasion, distant metastasis, and TNM stage were independent predictors of poor prognosis (Table 2). Lastly, the UCA1 expression level was evaluated in EC cells, which were normalized to GAPDH. QRT-PCR analysis showed all of the four EC cell lines expressed higher UCA1 than the normal cell line (Figure 1(f)). Together, these data suggest that increased UCA1 expression was presented in the malignant phenotypes of EC.

### 3.2. UCA1 Silencing Suppressed EC Cell Proliferation and Metastasis in EC Cells

**Figure 2:**
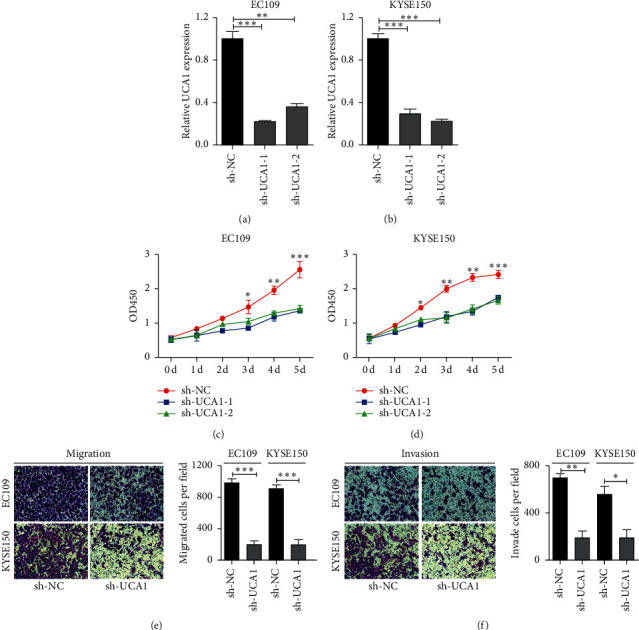
UCA1 plays an oncogenic role in EC cells. (a) and (b) UCA1 expression was detected by transfection with si-NC, si-UCA1-1, and si-UCA1-2 into EC109 and KYSE150 cells. (c) and (d) The effects of UCA1 knockdown on the proliferation of EC109 and KYSE150 cell lines. (e) and (f) Trans-well assays were performed to detect the effects of UCA1 silencing on cell migration and invasion in EC cells. Quantification of migrated and invaded cells was performed for five randomly selected fields (original magnification: 200x). Student's *t* test was used; ^*∗*^*p* < 0.05, ^*∗∗*^*p* < 0.01, and ^*∗∗∗*^*p* < 0.001.

To further assess the role of UCA1 in EC progression, we stably silenced UCA1 expression in EC109 and KYSE150 by small hairpin RNA (shRNA), as shown in Figures 2(a) and 2(b). And, sh-UCA1-1 was chosen for further study. In cell proliferation assays, UCA1 silencing obviously inhibited cellular viability of EC109 (Figure 2(c)) and KYSE150 (Figure 2(d)) cells compared with sh-NC groups. Next, we examined whether UCA1 affects the migratory ability of EC cells in vitro. Compared with sh-UCA1 groups, UCA1-deficient cells exhibited hindered migration (Figure 2(e)) and invasive abilities (Figure 2(f)) in both EC109 and KYSE150 cells. In brief, these findings meant that UCA1 deficient deferred the progression of ES.

### 3.3. MiR-203 Is a Direct Target of UCA

**Figure 3:**
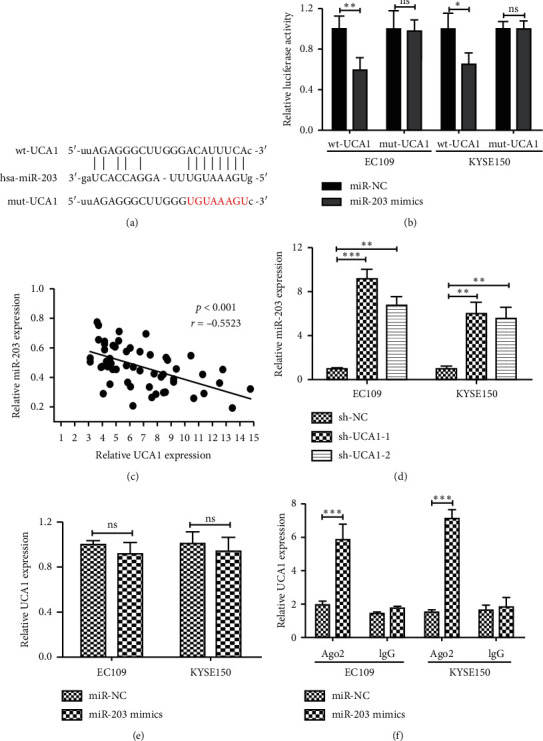
UCA1 acts as a molecular sponge for miR-203 in EC cells. (a) The predicted binding sites between UCA1 and miR-203 were predicted by microRNA.org and Starbase v2.0. UCA1 mutated at the putative binding site. (b) Dual-luciferase reporter assay was performed to verify miR-203 was the target gene of UCA1. (c) Pearson's correlation analysis between miR-203 and UCA1 expression in 60 EC samples (*r* = -0.5523, *p* < 0.001). (d) miR-203 expression was measured in UCA1 knockdown EC cells by qRT-PCR. (e) UCA1 expression was detected after miR-203 overexpression by qRT-PCR. (f) The levels of UCA1 bound to Ago2 or IgG measured by qRT-PCR after RIP in EC cells with miR-203 mimics and miR-NC; ^*∗*^*p* < 0.05, ^*∗∗*^*p* < 0.01, and ^*∗∗∗*^*p* < 0.001; ns, no significance.

Previous reports showed that lncRNAs could act as competing endogenous RNAs (ceRNAs) or molecular sponges to modulate miRNAs. Based on this ground, bioinformatics was conducted using microRNA.org (http://www.microrna.org) and Starbase v2.0 (http://starbase.sysu.edu.cn) to predict potential UCA1-miRNA interactions. The results showed that miR-203 contains a potential binding site for UCA1 (Figure 3(a)). To confirm the direct target between UCA1 and miR-203, dual-luciferase reporter assay was performed in EC cells. Our data showed that miR-203 mimics significantly reduced luciferase activity of UCA1-Wt but not of UCA1-Mut vectors in EC109 and KYSE150 cells (Figure 3(b); *p* < 0.05). Furthermore, we found that miR-203 expression was significantly negatively associated with the expression of UCA1 in EC samples (*r* = −0.523, *p* < 0.001, Figure 3(c)). We then examined the impact of UCA1 silencing on miR-203 in EC109 and KYSE150 cells. MiR-203 was significantly increased after UCA1 knockdown in EC cells (Figure 3(d)). However, miR-203 overexpression had no significant effect on the expression level of UCA1 (Figure 3(e)). Previous studies showed that Ago2 protein family plays a central role in RNA-induced silencing complex to bind to silence targeted mRNA [21]. Thus, RIP assays were used to confirm the interaction using AGO2 antibody. UCA1 was specifically enriched in the Ago2 pellet of miR-203 overexpressed cells (Figure 3(f)). Thus, miR-203 was a direct inhibitory target of UCA1 in EC.

### 3.4. UCA1 Promotes HK2 Expression by Sponging miR-203

**Figure 4:**
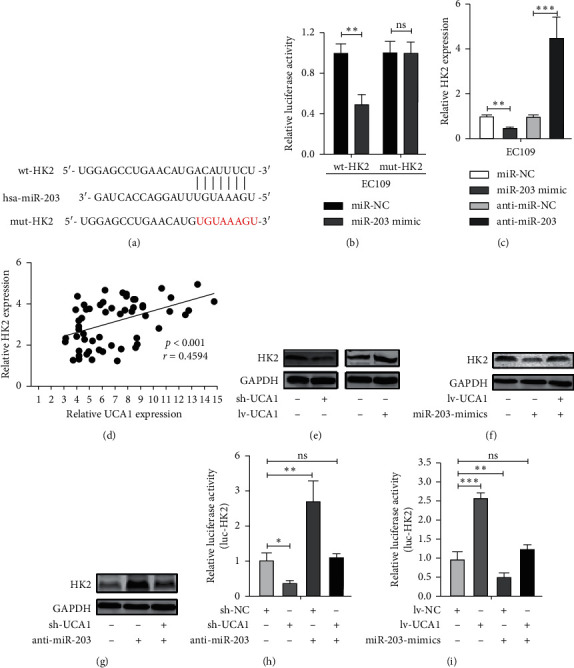
UCA1 reverses the inhibitory effect of miR-203 on HK2. (a) Predicted miR-203 binding sites of HK2 and the corresponding mutant sequence. (b) The miR-203 targets the 3'UTR of HK2 analyzed by the luciferase reporter assays in EC109 cell. (c) The expression levels of HK2 decreased after miR-203 overexpression and increased by the miR-203 inhibitor. (d) Positive correlation between UCA1 and HK2 expression in EC tissues detected by Pearson's correlation analysis (*r* = 0.4594, *p* < 0.001). (e) Downregulated UCA1 inhibited the HK2 expression and ectopic expression of UCA1 (lv-UCA1) and increased the HK2 expression in EC109 cells. (f) Upregulation of UCA1 could reverse the inhibit effect of miR-203 on HK2 expression. (g) UCA1 silencing could reverse the miR-203 inhibitor-mediated upregulation of HK2. (h) Luciferase activity in EC109 cells transfected with luciferase reporters containing HK2 3'-UTR following transfection with sh-NC, sh-UCA1, anti-miR-203 + sh-NC, or anti-miR-293 + sh-UCA1. (i) Luciferase activity in EC109 cells transfected with luciferase reporters containing HK2 3'-UTR following transfection with lv-NC, lv-UCA1, miR-203-mimics + lv-NC, or miR-203-mimics + lv-UCA1. Data are shown as mean ± SEM; ^*∗∗*^*p* < 0.01 and ^*∗∗∗*^*p* < 0.001.

HK2 (hexokinase), one of the three key rate-limiting enzymes of glycolysis, could be involved in tumor progression by miRNA adjusting [22]. Hence, to investigate whether miR-203 targets HK2 in ES cells, the putative recognition sequence between miR-203 and the 3′UTR of HK2 was determined using microRNA.org (Figure 4(a)). Following this, luciferase reporter assay demonstrated that miR-203 significantly suppressed the luciferase activity of report gene containing wild-type of HK2 (wt), while there was no effect on the mutated 3'-UTR of HK2 (mut) in EC cells (Figure 4(b)). Besides, the inhibiting effect of miR-203 on HK2 expression was verified in EC109 cells (Figure 4(c)).

Next, given that miR-203 was capable of targeting both UCA1 and HK2, we tested whether UCA1 could mediate HK2 expression through competitively sequestering miR-203. Firstly, the relationship between HK2 and UCA1 mRNA expression was analyzed using Pearson's correlation analysis in EC tissues. The result showed that a significant positive correlation existed between UCA1 and HK2 (*r* = 0.4594, *p* < 0.001) (Figure 4(d). Besides, positive regulation between UCA1 and HK2 was verified in EC109 cells. UCA1 knockdown could decrease the HK2 protein expression, and ectopic expression of UCA1 (lv-UCA1) increased the levels of HK2 (Figure 4(e)). Then, we determined whether UCA1 regulates HK2 through its regulatory role on miR-203. The results showed that UCA1 overexpression attenuated the depress effect on the protein expression levels of HK2 induced by miR-203 mimics (Figure 4(f)). Besides, the miR-203 inhibitor-mediated upregulation of HK2 was apparently reversed by UCA1 silencing (Figure 4(g)). To verify the phenomenon above, a luciferase reporter gene fused with wild-type of HK2 was constructed. UCA1 silencing significantly reduced the luciferase activity, while the miR-203 inhibitor relieved this decrease (Figure 4(h)). In contrast, UCA1 overexpression increased the reporter activity, while miR-203 mimics attenuated this increase (Figure 4(i). And, there was no significant change in luciferase activity of the control luciferase reporter gene containing no fused gene (data not shown) in EC109 cells. Taken together, these results suggest that, by binding miR-203, UCA1 acts as a ceRNA-targeting HK2, modulating the HK2 expression.

### 3.5. UCA1 Promotes Aerobic Glycolysis via HK2

**Figure 5:**
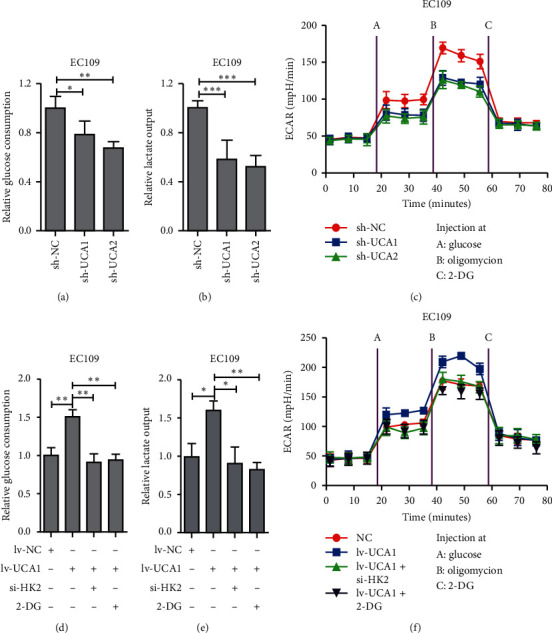
UCA1 regulated glycolysis through HK2. (a) Glucose consumption measured in EC109 cells transfected sh-UCA1. (b) Lactate production measured in EC109 cells transfected sh-UCA1. (c) Extracellular acid ratio (ECAR) measured in EC109 cells transfected sh-UCA1. (d) Glucose consumption measured in UCA1-overexpressing EC109 cells transfected with HK2 siRNA or 2-DG. (e) Lactate production measured in UCA1-overexpressing EC109 cells transfected with HK2 siRNA or 2-DG. (f) Extracellular acid ratio (ECAR) in UCA1-overexpressing EC109 cells transfected with HK2 siRNA or 2-DG. Student's *t* test was used; ^*∗*^*p* < 0.05, ^*∗∗*^*p* < 0.01, and ^*∗∗∗*^*p* < 0.001.

As known to us, hexokinase 2 (HK2), a key mediator of glycolysis enzyme, was involved in multicancer [23, 24]. Furthermore, cancer cell is characterized by elevated metabolism in glycolysis and lactate production had been identified as the characteristic of tumor cells, even in the presence of abundant oxygen, which was termed as Warburg effect or aerobic glycolysis [25]. In view of this, we firstly examined the relationship between UCA1 and aerobic glycolysis in EC109 cells. Both glucose uptake and lactate output were marked decreased in UCA1-knockdown EC109 cells compared with those in the control group (Figures 5(a) and 5(b)). Meanwhile, significantly weakened extracellular acid rate (ECAR) was detected in UCA1-knockdown EC109 cells, indicating that UCA1 affects the capability of glucose metabolism (Figure 5(c)). We next sought to explore the metabolic recombination process by which UCA1 mediated the expression of HK2. The results showed that HK2 downregulation obviously attenuated the increase in glucose uptake and lactate production induced by UCA1 overexpression (Figures 5(d) and 5(e)). A similar trend emerged when treated with 2-Deoxy-D-glucose (2-DG), a HK2 inhibitor (Figures 5(d) and 5(e)). And, HK2 downregulated or treatment with 2-DG obviously abolished the increase in ECAR induced by UCA1 overexpression (Figure 5(f)). Collectively, these data indicated that UCA1-induced aerobic glycolysis was HK2 dependent.

## 4. Discussion

In this study, we focused our research on the underlying molecular mechanism of UCA1 in EC. Here, we showed that UCA1 was upregulated in EC with malignant phenotype and unsatisfactory prognosis. In vitro study also demonstrated that UCA1 could mediate the EC cells proliferation and metastasis. All of this proved UCA1 worked as an oncogene, similar to other tumors [14–18].

Following, the molecular mechanism how UCA1 involves in EC need further investigation. Abundant studies have discovered that lncRNA can act as ceRNAs via miRNA-binding sites to regulate target mRNA levels [26]. UCA1 has been reported to contain the binding site of many miRNAs to be participated in multi tumors. Interaction between UCA1 and miR-204-5p enhances cell growth and 5-fluorouracil resistance in colorectal cancer [27]. In melanomas, Interaction between UCA1 and miR-507 inhibit FOXM1 protein degradation to enhance cell proliferation, invasion, and G0/G1 cell cycle arrest [28]. As predicted by online tools, UCA1 possesses putative binding site of miR-203. And luciferase activity assay confirms this. Furthermore, negatively association was found between miR-203 and UCA1 expression in EC tissues. Further study demonstrated that UCA1 knockdown could suppress miR-203 expression in EC cells, while no significantly effect of miR-203 mimics on UCA1 expression occurred. In RNA-IP assay, we found that UCA1 was significantly pulled down in miR-203 overexpressing cells. Inspired by this, we proposed that UCA1 could directly target miR-203 in EC cancer. Previous studies confirmed that miR-203 worked as tumor suppressor in various kinds of cancers, including EC [29]. Therefore, UCA1 may function, in part, through inhibition of miR-203 in EC. As a further matter, other miRNAs may also be target regulated by UCA1; hence, further study should be designed to figure out the potential miRNAs.

Following this, bioinformatics analysis and luciferase reporter confirmed that miR-203 worked as tumor suppressor through binding HK2 in EC. Given that miR-203 was capable of targeting both UCA1 and HK2, we tested whether UCA1 could mediate HK2 expression. Firstly, positively association was found between UCA1 and HK2 expression in EC tissues. Meanwhile, downregulated UCA1 and ectopic expression of UCA1 could inhibit and enhance HK2 expression, respectively. Furthermore, we showed that UCA1 overexpression could attenuate the degradation of HK2 induced by miR-203 mimics and UCA1 silencing could reverse miR-203 inhibitor-mediated upregulation of HK2. To reinforce the hypothesis, a luciferase reporter containing 3'-UTR of HK2 was constructed. In consistent to our hypothesis, UCA1 could obviously change the luciferase activity as well as reverse the miR-203 effect on the luciferase activity. Thus, these results showed that HK2 is a direct downstream target of UCA1 by sponging to miR-203 in EC cells.

HK2, a vital metabolic enzyme of glycolysis, promote glucose uptake in cells and facilitate the Warburg effect [30]. Inspired by this, we test the effect of UCA1 on the aerobic glycolysis for the first time in EC cells. Here, we showed that UCA1 silencing markedly decreased the glucose uptake and lactate output in EC109 cells. Meanwhile, significantly weakened ECAR was detected in UCA1-knockdown EC109 cells, which indicated that UCA1 affects the capability of glucose metabolism. Further study revealed that inhibition of HK2 activity by interfering HK2 expression or inhibitor could attenuate the acceleration of UCA1 overexpression on glucose uptake, lactate output, and ECAR. Therefore, we conclude that UCA1 exerts a critical role in the mediation of glucose metabolism in EC cells.

## 5. Conclusions

**Figure 6:**
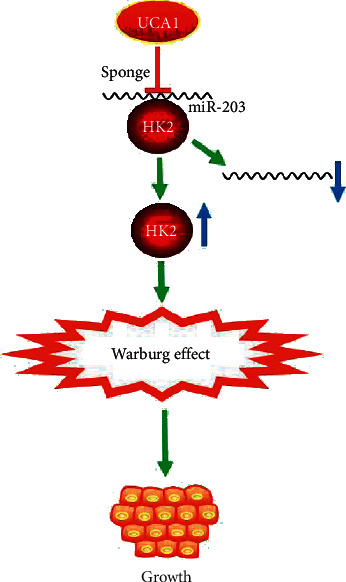
Schematic diagram showing the role of UCA1 in the progression of EC.

Overall, our study verified that UCA1 exert oncogene role via enhancing glycolysis in EC (Figure 6). UCA1 promoted glycolysis by sequestering miR-203 and relieving the inhibitory effect of miR-203 on HK2, thus increasing the level of HK2 and contributing to Warburg effect. Our study provides novel insights into the molecular mechanisms underlying glycolysis in tumorigenesis.

## Figures and Tables

**Table 1:** Clinicopathological correlation of UCA1 expression in 110 EC patients.

Clinicopathological feature	Total	Expression of UCA1		*p* value (*χ*^2^ test)
		Low	High
Age (years)				
≤60	56	29	27	0.703
>60	54	26	28	

Gender				
Male	90	45	45	1.000
Female	20	10	10	

Tumor size				
≤3 cm	45	28	17	**0.033**
>3 cm	65	27	38	

Smoking status				
Yes	67	31	36	0.329
No	43	24	19	

Alcohol status				
Yes	65	25	40	**0.004**
No	45	30	15	

Tumor location				
Lower	35	15	20	0.406
Middle	61	34	27	
Upper	14	6	8	

TNM stage				
I + II	47	39	8	**<0.001**
III + IV	63	16	47	

Lymphatic invasion				
Yes	56	11	45	**<0.001**
No	54	40	10	

Distant metastasis				
Yes	26	5	21	**0.001**
No	84	50	34	

Differentiation				
Low	35	20	15	0.282
Moderate	55	28	27	
Well	20	7	13	

The bold number represents the *p* values with significant differences.

**Table 2:** Univariate analysis of prognostic parameters for survival in EC patients.

Prognostic parameter	Univariate analysis			Multivariate analysis		
	HR	95% CI	*p* value	HR	95% CI	*p* value
Expression of UCA1 (low vs. high)	3.921	2.323–6.618	**<0.001**	2.161	1.132–4.127	**0.020**
Age (≤60vs. > 60)	1.287	0.802–2.065	0.296			
Gender (male vs. female)	0.779	0.439–1.383	0.394			
Tumor size (≤3 cm vs > 3 cm)	1.437	0.874–2.361	0.153			
Smoking status (yes vs. no)	0.942	0,582–1.523	0.806			
Alcohol status (yes vs. no)	1,433	0.833–2,360	0.144			
Tumor location (lower, middle, vs. upper)	1.082	0.741–1.581	0.682			
Lymphatic invasion (yes vs. no)	2.845	1.596–5.071	**<0.001**	19.646	2.562–150.643	**0.004**
Distant metastasis (yes vs. no)	5.971	3.496–10.198	**<0.001**	7.035	3.608–13.717	**<0.001**
Differentiation (low, moderate, vs. well)	1.059	0.749–1.498	0.746			
TNM stage (I + II and III + IV)	1.508	1.164–1.953	**0.002**	0.069	0.009–0.552	**0.012**

HR: hazard ratio; CI: confidence interval. The bold number represents the *p* values with significant differences.

## Data Availability

The data used to support the findings of this study are available from the corresponding author upon request.
